# Platelet adhesion assessed by PFA-100 is not linked to progression of ACLD

**DOI:** 10.1016/j.jhepr.2023.100934

**Published:** 2023-10-12

**Authors:** Lorenz Balcar, Benedikt Simbrunner, Rafael Paternostro, Mathias Jachs, Lukas Hartl, Georg Semmler, Benedikt Silvester Hofer, Albert Friedrich Stättermayer, Matthias Pinter, Ton Lisman, Michael Trauner, Peter Quehenberger, Thomas Reiberger, Bernhard Scheiner, Mattias Mandorfer

**Affiliations:** 1 Division of Gastroenterology and Hepatology, Department of Internal Medicine III, Medical University of Vienna, Vienna, Austria; 2 Vienna Hepatic Hemodynamic Lab, Division of Gastroenterology and Hepatology, Department of Internal Medicine III, Medical University of Vienna, Vienna, Austria; 3 Surgical Research Laboratory and Section of Hepatobiliary Surgery and Liver Transplantation, Department of Surgery, University of Groningen, University Medical Center Groningen, Groningen, The Netherlands; 4 Department of Laboratory Medicine, Medical University of Vienna, Vienna, Austria

**Keywords:** Platelet, Cirrhosis, Portal hypertension, Acute-on-chronic liver failure

## Abstract

**Background & Aims:**

Increased aggregation of individual platelets upon activation, as assessed by whole blood aggregometry standardised to platelet count (PLT), has recently been linked to progression of advanced chronic liver disease (ACLD). Moreover, changes in primary haemostasis have been implicated in bleeding and thrombosis in patients with ACLD.

We aimed (i) to identify the determinants of the primary haemostatic capacity – as assessed by Platelet Function Analyzer 100 (PFA-100) (‘*in vitro* bleeding time’) – in patients with ACLD and (ii) to investigate its potential association with clinical outcomes.

**Methods:**

PFA-100 was performed in 688 patients with ACLD undergoing hepatic venous pressure gradient measurement. Hepatic decompensation and liver-related death as well as bleeding and thrombosis were the outcomes of interest.

**Results:**

Sixty-three percent of patients had a PFA-100 collagen/epinephrine closure time (CT) of >150 s (*i.e.* prolonged). PLT and haematocrit were the main determinants of CT, whereas it was not impacted by von Willebrand factor antigen. Mirroring the increasing prevalence/severity of thrombocytopaenia and anaemia, we observed a progressive prolongation of CT (*i.e.* decreased primary haemostatic capacity) with more advanced disease, as indicated by clinical stage, Child–Turcotte–Pugh score, United Network for Organ Sharing model for end-stage liver disease (2016) score, and hepatic venous pressure gradient. Although increased CT (*i.e*. decreased primary haemostatic capacity) was associated with an increased risk of hepatic decompensation/liver-related death, these associations were less consistent after adjusting/correcting for PLT/haematocrit and established prognostic indicators. Finally, CT was not associated with the incidence of major bleedings or thromboses.

**Conclusions:**

These findings do not support the hypothesis that increased platelet adhesion – assessed *in vitro* under shear stress by PFA-100 – promotes ACLD progression.

**Impact and implications:**

The potential of platelets to aggregate in the bloodstream may be increased in patients with advanced chronic liver disease. Platelet Function Analyzer 100 (PFA-100), a blood test reflecting *in vitro* bleeding time, might be suggestive of an impaired primary clot forming capacity. In our study, we could show that PFA-100 results were not linked to bleeding/thrombotic events. Our findings do not support the hypothesis that an increased adhesion of platelets (assessed by PFA-100) might lead to a disease progression in patients with advanced chronic liver disease.

## Highlights

• PFA-100 results are commonly suggestive of an impaired primary haemostatic capacity.
• Long *in vitro* bleeding time tended to increase risks of adverse liver-related outcomes.
• PFA-100 results were not associated with bleeding/thrombotic events.
• Our findings do not support the hypothesis that increased platelet adhesion – assessed *in vitro* under shear stress by PFA-100 – promotes ACLD progression.

## Graphical abstract

**Figure:**
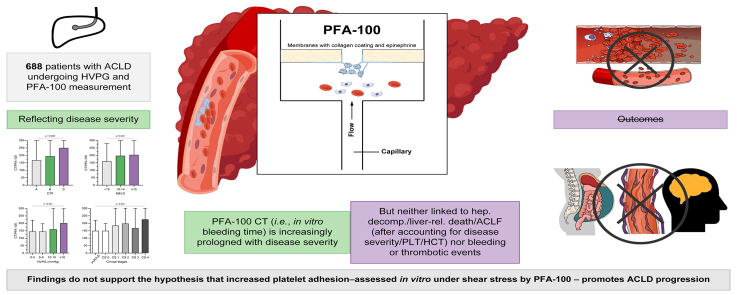

## Introduction

Platelets are essential for haemostasis as they form an initial platelet plug sealing the damaged vessel wall and subsequently support the coagulation cascade. In patients with advanced chronic liver disease (ACLD), thrombocytopaenia – beyond other mechanisms – reflects severity of portal hypertension/hepatic dysfunction^1^ and thus, increases in frequency/severity with disease progression. In contrast to historic belief, the aggregatory potential of platelets may be substantially increased in patients with ACLD compared with healthy controls, independent of thrombocytopaenia severity.^2^ The hyper-reactivity of individual platelets upon activation by agonists, as assessed by whole blood aggregometry (more specifically Multiplate; Roche Diagnostics, Mannheim, Germany) standardised to platelet count (PLT), has recently been linked to progression of ACLD.^3^ However, whether the standardisation for PLT applied in this study is fully justified remains unclear, as PLT-standardised Multiplate test results showed substantial intra-individual variability across different PLT levels in the experimental study that established this ratio. In addition to liver disease progression, changes in primary haemostasis have also been implicated in bleeding and thrombosis in patients with ACLD.^4^

In contrast to Multiplate, Platelet Function Analyzer 100 (PFA-100) assesses platelet plug formation in flowing blood.^5^ Whole blood is added to a cuvette containing a small opening lined with collagen and either epinephrine or adenosine diphosphate (ADP). The blood flows through the opening under high shear stress, which will close when sufficient platelets have aggregated in response to adhesion to collagen and subsequent aggregate formation stimulated by epinephrine or ADP. The time until occlusion (and thereby termination of blood flow) is defined as the closure time (CT). PFA-100 has proven its utility in evaluation of (severe) defects of platelet function, von Willebrand disease, and monitoring of aspirin therapy.^6^ Moreover, short CT values have been linked to venous thromboembolism in the RETROVE trial.^7^ However, the test is sensitive for thrombocytopaenia and anaemia, which prolong the CT,^6^ and thus, the interpretation of PFA-100 test results under these conditions or in patients with ACLD may not be straightforward. In the context of our study, PFA-100 results may be interpreted as a global test for the primary haemostatic capacity, that is, an ‘*in vitro* bleeding time’.

Because data on PFA-100 in patients with ACLD are scarce, the objective of this study was (i) to identify the determinants of the primary haemostatic capacity – as assessed by PFA-100 (‘*in vitro* bleeding time’) – in patients with ACLD and (ii) to investigate its potential association with clinical outcomes.

## Patients and methods

### Study design and patients

We performed a retrospective, single-centre cohort study in patients with ACLD undergoing hepatic venous pressure gradient (HVPG) measurement at the Vienna Hepatic Hemodynamic Lab between September 2003 and December 2020. Inclusion criteria were (i) HVPG ≥6 mmHg and (ii) availability of information on PFA-100 results. Patients were excluded if any of the following criteria were present: history of orthotopic liver transplantation, underlying non-ACLD aetiology, any active malignancies, presence of portal vein thrombosis (PVT), current anticoagulation and/or antiplatelet therapy, evidence of bacterial infection, acute-on-chronic liver failure (ACLF) at study inclusion, unreliable HVPG, or missing information on clinical follow-up.

### Clinical stages of ACLD

Patients were classified according to previously defined prognostic or clinical stages (CSs). CSs were defined according to D’Amico *et al.*^8^

### HVPG measurement

HVPG measurement was performed in the absence of non-selective betablocker therapy and in adherence to a standard operating procedure.^9^ Briefly, a catheter introducer sheath was inserted into the right internal jugular vein under local anaesthesia and ultrasound guidance. Subsequently, a hepatic vein was cannulated using a dedicated balloon catheter,^10^ and the free and wedged hepatic venous pressures were obtained at least as triplicate measurements, according to recent Baveno VII guidelines.^11^

### Measurement of key laboratory parameters

Routine laboratory tests and PFA-100 (Siemens Healthcare Diagnostics, Erlangen, Germany) measurements were performed using blood samples obtained via a central venous line (*i.e.* the sideport of the catheter introducer sheath) at the time of HVPG measurement by the ISO-certified Department of Laboratory Medicine of our institution using commercially available methods that are applied in clinical routine. PFA-100 measurements were performed in adherence to the manufacturer’s instructions. In the present study, only CT with collagen/epinephrine as agonist was considered for analysis, as testing with the collagen/ADP cartridge was only performed in those in whom the more sensitive collagen/epinephrine CT^6^ was prolonged. PLT/haematocrit-corrected CT values were calculated using a previously established formula.^5^

### Bleeding and thrombotic events

To evaluate the association between CT and clinical outcomes, we assessed bleeding and thrombotic events during follow-up. Although relevant thrombotic as well as major bleeding events lead to medical contact, patients with minor bleeding episodes may commonly not be present at the hospital, and thus, these events are likely to be underestimated in a retrospective study. Therefore, we focused our analyses on major bleeding as well as arterial/venous thrombotic events. According to international recommendations,^12^^,^^13^ the following bleeding events were considered major: fatal bleeding events, symptomatic bleeding episodes in a critical area or organ (*i.e.* intracranial, intraspinal, intraocular, retroperitoneal, intra-articular or pericardial, or intramuscular with compartment syndrome), bleeding events with an associated decrease in haemoglobin level of ≥2 g/dl, or bleeding episodes leading to transfusion of two or more units of packed red blood cells. Bleeding events were further classified as being related to portal hypertension or not. In addition, we reviewed the patients’ health records for the development of thrombotic events. The following events were considered relevant for this study: non-tumoural PVT, deep vein thrombosis, superficial vein thrombosis, and pulmonary embolism as well as myocardial infarction and stroke.

### Statistical analysis

All statistical analyses were performed using IBM SPSS Statistics 27 (IBM, New York, NY, USA), R 4.3.1 (R Core Team, R Foundation for Statistical Computing, Vienna, Austria), or GraphPad Prism 8 (GraphPad Software, Boston, MA, USA). Categorical variables were reported as absolute (n) and relative frequencies (%), whereas continuous variables as mean ± SD or median (IQR), as appropriate. Student’s *t* test was used for group comparisons of normally distributed variables and the Mann–Whitney *U* test for non-normally distributed variables.

Univariable and multivariable linear regressions were calculated to investigate factors associated with CT.

Time-dependent event rates were obtained using the reverse Kaplan–Meier method. Univariable and multivariable Cox regression analyses were performed to evaluate parameters independently associated with the events of interest. The impact of PFA-100 results (displayed per 10 s) on hepatic decompensation/liver-related death and liver-related death alone were assessed using competing risk analyses considering date of removal/suppression of the primary aetiological factor (initiation of antiviral therapy/reported alcohol abstinence, as defined by Baveno VII), requirement of liver transplantation, or non-liver-related death, as competing risks. Therefore, Fine and Gray competing risk regression models (cmprsk: subdistribution analysis of competing risks; https://CRAN.R-project.org/package=cmprsk)^14^^,^^15^ were calculated. Baseline characteristics that are known to affect CT values (*i.e.* PLT and haematocrit) and may have some prognostic implications as well as parameters that we considered of particular importance for the endpoint of interest (*i.e.* age, indicators of hepatic dysfunction, and HVPG) were included into multivariable competing risk models as covariables. The Child–Turcotte–Pugh (CTP) and United Network for Organ Sharing (UNOS) model for end-stage liver disease (MELD) (2016) scores have significant overlap in terms of included variables. Therefore, we generated separate models with either CTP or UNOS MELD (2016) scores. To compare PFA-100 results with other clinical measures/scores (*i.e.* HVPG and UNOS MELD [2016]), we calculated time-dependent area under the receiver operating characteristic curves (AUROCs).

Furthermore, univariable and multivariable competing risk regression analyses with the development of ACLF and liver-related death as outcomes of interest (excluding patients with ACLF at baseline; same competing risks as shown above) were performed.

Finally, bleeding and thrombotic events were evaluated during follow-up. Univariable competing risk regression analyses for any major bleedings, major portal-hypertensive bleedings, and any non-malignant thromboses with initiation of antiplatelet therapy, anticoagulation, or death as competing risks were calculated.

The level of significance was set at a two-sided *p* value of <0.05.

### Ethics

The study was conducted in accordance with the principles of the Declaration of Helsinki and was approved by the local ethics committee. The requirement of written informed consent for this retrospective study was waived by the ethics committee.

## Results

### Patient characteristics

**Fig. 1:**
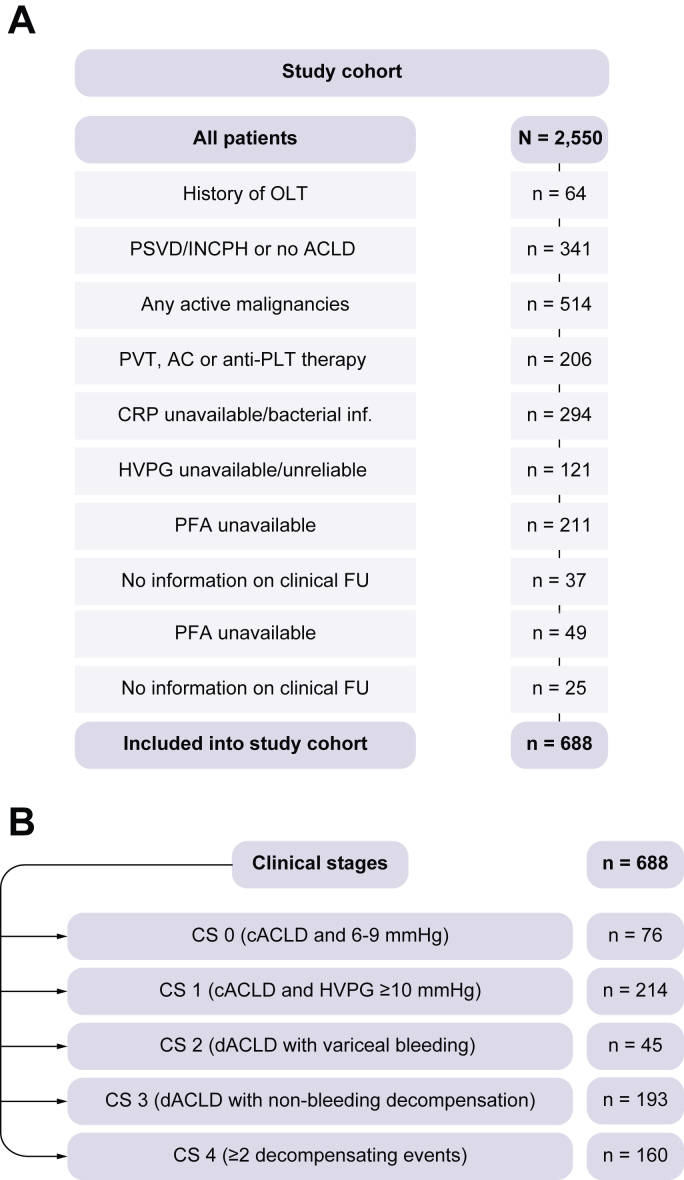
Study cohort. (A) Patient flowchart including inclusion and exclusion criteria. (B) Number of patients within different CS. AC, anticoagulation; ACLD, advanced chronic liver disease; cACLD, compensated ACLD; ACLF, acute-on-chronic liver failure; CRP, C-reactive protein; CS, clinical stage; dACLD, decompensated ACLD; FU, follow-up; HVPG, hepatic venous pressure gradient; INCPH, idiopathic non-cirrhotic portal hypertension; LSM, liver stiffness measurement; OLT, orthotopic liver transplantation; PFA, Platelet Function Analyzer 100; PLT, platelet; PSVD, porto-sinusoidal vascular disorder; PVT, portal vein thrombosis.

Overall, 2,550 individual patients underwent HVPG measurement at the Vienna General Hospital during the study period. After inclusion and exclusion criteria were applied, 688 patients were included in this study (Fig. 1).

Mean age was 53.2 ± 11.3 years, and most patients were male (n = 459, 67%) (Table S1). The main aetiologies of liver disease were alcohol-related liver disease (n = 281, 41%) and viral hepatitis (n = 259, 38%). At baseline, 290 (42%) patients were considered compensated, whereas n = 398 (58%) had already experienced decompensation (mostly stable patients with decompensation, n = 328, 48%). Mean UNOS MELD (2016) was 13 ± 5 points, and mean HVPG was 17 ± 6 mmHg. Median PLT was 102 (IQR 69–142) G/L, mean haematocrit was 34.1 ± 5.7%, and mean plasma von Willebrand factor antigen (VWF–Ag) was 324%. Sixty-three percent of patients (n = 435) had a PFA-100 collagen/epinephrine CT of >150 s (*i.e.* prolonged).

More detailed information on baseline characteristics is displayed in Table S1.

### PFA-100 increases with liver disease severity

**Fig. 2:**
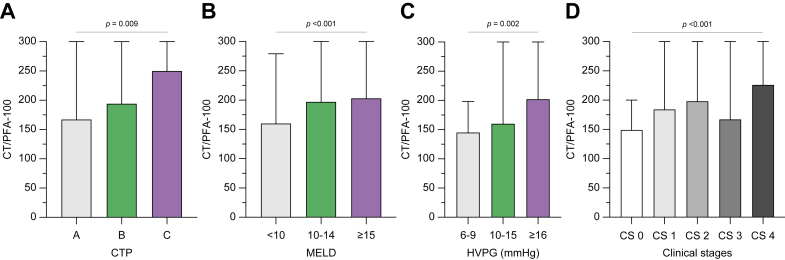
CT/PFA-100 across disease severity strata. Comparison of CT/PFA-100 according to (A) CTP, (B) UNOS MELD (2016) score, and (C) HVPG strata as well as (D) CSs. CT/PFA-100 levels were plotted as median (IQR) and compared using the Mann–Whitney *U* test. CS, clinical stage; CT, closure time; CTP, Child–Turcotte–Pugh; HVPG, hepatic venous pressure gradient; PFA-100, Platelet Function Analyzer-100; UNOS MELD (2016), United Network for Organ Sharing model for end-stage liver disease (2016).

As shown in Fig. 2 and Table S2, CT increased with liver disease severity, that is, CTP (*p* = 0.009) and UNOS MELD (2016) scores (*p* <0.001), as well as HVPG strata (*p* = 0.002) and CS (*p* <0.001). Interestingly, there was also a trend-wise increase in CT values when substratifying patients with decompensation according to the clinical course of decompensation (*i.e.* stable *vs*. unstable *vs*. pre-ACLF *vs*. ACLF; *p* = 0.202).

However, Tables S3 and S4 depict that not only CT increased but also PLT and haematocrit levels decreased across disease severity, portal hypertension strata, or CS.

### Adjusted and unadjusted analyses of factors associated with CT

In univariable analyses, CT was directly associated with severity of liver disease (CTP score: unstandardised regression coefficient [B] = 5.295, *p* <0.001; UNOS MELD [2016]: B = 2.216, *p* <0.001) and portal hypertension severity (HVPG: B = 2.411, *p* <0.001) (Table 1). In addition, there was a positive association with age, diabetes, arterial hypertension, and decompensation status. Finally, CT was negatively associated with male sex, hypertriglyceridaemia, albumin levels, and PLT/haematocrit levels.

**Table 1:** Simple and multiple linear regression analysis of factors associated with CT/PFA-100 including, among other parameters, either CTP score, sodium, and creatinine (model 1) or UNOS MELD (2016) score, CS, and albumin (model 2).

Patient characteristics	Univariable		Model 1 (including CTP score, sodium, and creatinine)		Model 2 (including MELD, CS, and albumin)	
	B	*p* value	B	*p* value	B	*p* value
Age (years)	0.635	**0.016**	0.276	0.350	0.319	0.262
Male sex	-14.016	**0.027**	-5.122	0.442	-4.546	0.484
BMI (kg × m^-2^)	1.000	0.075	0.438	0.485	0.648	0.295
Overweight∗	6.411	0.285	—	—	—	—
Obesity†	11.566	0.111	—	—	—	—
Prediabetes‡	6.188	0.425	—	—	—	—
Diabetes§	16.297	**0.032**	-1.154	0.875	-1.019	0.889
Arterial hypertension¶	11.876	0.059	16.027	**0.020**	14.583	**0.034**
Hypertriglyceridaemia∗∗	-33.893	**0.004**	-15.806	0.192	-19.207	0.111
Hypercholesterolaemia††	-7.567	0.431	—	—	—	—
HDL below threshold‡‡	2.902	0.657	—	—	—	—
Statin use	0.611	0.970	—	—	—	—
Hepatic steatosis§§	5.866	0.383	—	—	—	—
CTP score (point)	5.295	**<0.001**	-0.176	0.929	—	—
UNOS MELD (2016) score (point)	2.216	**<0.001**	—	—	0.971	0.203
HVPG (mmHg)	2.411	**<0.001**	0.251	0.680	0.344	0.577
dACLD (CS 2 to CS 4)	11.322	0.061	—	—	-19.552	**0.009**
Platelet count (G × L^-1^)	-0.404	**<0.001**	-0.368	**<0.001**	-0.381	**<0.001**
Haematocrit (%)	-4.523	**<0.001**	-4.920	**<0.001**	-5.520	**<0.001**
Sodium (mmol × L^-1^)	0.630	0.378	1.184	0.164	—	—
Creatinine (mg × dl^-1^)	5.651	0.625	-0.466	0.969	—	—
Albumin (g × L^-1^)	-1.410	**0.006**	—	—	0.754	0.222
CRP (mg × dl^-1^)	-6.351	0.218	—	—	—	—
VWF-Ag (%)	0.004	0.870	—	—	—	—

Values of *p* in bold denote *p* <0.05.

ACLD, advanced chronic liver disease; CRP, C-reactive protein; CS, clinical stage; CTP, Child–Turcotte–Pugh; dACLD, decompensated ACLD; HbA_1c_, haemoglobin A_1c_; HVPG, hepatic venous pressure gradient; UNOS MELD (2016), United Network for Organ Sharing model for end-stage liver disease (2016); VWF-Ag, von Willebrand factor antigen.

∗ BMI ≥25 kg × m^-2^.

† BMI ≥30 kg × m^-2^.

‡ Fasting blood glucose 100–125 mg × dl^-1^ and HbA_1c_ 5.7–6.4%.

§ Fasting blood glucose >125 mg × dl^-1^, HbA_1c_ ≥6.5%, or antidiabetic medication.

¶ Blood pressure >140/90 mmHg or antihypertensive medication.

∗∗ Triglycerides >150 mg × dl^-1^.

†† Total cholesterol >200 mg × dl^-1^.

‡‡ <35 mg × dl^-1^ for males and <39 mg × dl^-1^ for females.

§§ Biopsy-proven, controlled attenuation parameter >248 dB × m^-1^ or diagnosed by ultrasound.

However, after multivariable adjustment for several parameters including either CTP score, sodium, and creatinine (model 1) or UNOS MELD (2016) and decompensated cirrhosis (CS 2 to CS 4) (model 2), arterial hypertension (model 1: B = 16.027, *p* = 0.020; model 2: B = 14.583, *p* = 0.034), decompensation status (model 2: B = -19.552, *p* = 0.009), and PLT (model 1: B = -0.368, *p* <0.001; model 2: B = -0.381, *p* <0.001) and haematocrit levels (model 1: B = -4.920, *p* <0.001; model 2: B = -5.520, *p* <0.001) were the only parameters associated with CT. Notably, VWF-Ag was not associated with CT. Data on ‘sophisticated’ biomarkers of bacterial translocation/systemic inflammation (*i.e.* lipopolysaccharide-binding protein, IL-6, and procalcitonin) were available in 202/688 patients (29%) and can be found in Tables S5–S8. The main disease severity indices (HVPG, UNOS MELD [2016], and CTP) as well as C-reactive protein (CRP) were not significantly different between the two groups, despite considerable sample size. Notably, none of these markers were linked to PFA-100/CT.

### Association of CT and hepatic decompensation and/or liver-related death

During follow-up, 182 deaths (26%) were considered liver-related, and 61 (9%) were considered non-liver-related. Overall, 67 patients (10%) underwent liver transplantation, 257 patients (37%) experienced first/further decompensation, and 130 patients developed ACLF (19%).

CT not only increased with liver disease severity in cross-sectional analyses but was also longitudinally associated with hepatic decompensation and liver-related death (subdistribution hazard ratio [SHR] 1.02, 95% CI 1.01–1.03, *p* <0.001) (Table 2). However, after adjusting for determinants of CT beyond platelet function (PLT and haematocrit in model 1) as well as known prognostic indicators (age, HVPG, haematocrit, and CRP, as well as CTP score, sodium, and creatinine levels in model 2 and UNOS MELD [2016], CS, and albumin levels in model 3), its association was lost in multivariable models: model 1, adjusted SHR (aSHR) 1.00 (95% CI 0.99–1.02), *p* = 0.970; model 2, aSHR 1.00 (95% CI 0.99–1.02), *p* = 0.690; and model 3, aSHR 1.01 (95% CI 0.99–1.02), *p* = 0.520 (Table 2). Similar results were found with liver-related death as the outcome of interest (Table 3). Once more, associations were lost after adjusting for relevant variables. Alternatively, when correcting the CT for thrombocytopaenia and anaemia using a previously published formula considering PLT/haematocrit,^5^ longer corrected CT was even independently associated with increased risks of hepatic decompensation and liver-related death (Tables S9 and S10).

**Table 2:** Univariable and multivariable competing risk regression analyses of factors associated with hepatic decompensation/liver-related death.

Patient characteristics	Univariable		Model 1 (including PLT and haematocrit)		Model 2 (including CTP score, sodium, and creatinine)		Model 3 (including MELD, CS, and albumin)	
	SHR (95% CI)	*p* value	aSHR (95% CI)	*p* value	aSHR (95% CI)	*p* value	aSHR (95% CI)	*p* value
Age (years)	1.02 (1.01–1.03)	**<0.001**	—	—	1.02 (1.01–1.03)	**0.003**	1.02 (1.01–1.03)	**0.002**
HVPG (mmHg)	1.08 (1.06–1.10)	**<0.001**	—	—	1.05 (1.02–1.07)	**<0.001**	1.03 (1.01–1.06)	**0.005**
CTP score								
A	1		—	—	1		—	—
B	2.34 (1.88–2.91)	**<0.001**	—	—	1.42 (1.09–1.86)	**0.010**	—	—
C	3.09 (2.30–4.15)	**<0.001**	—	—	1.62 (1.10–2.38)	**0.014**	—	—
UNOS MELD (2016) score (point)	1.06 (1.04–1.08)	**<0.001**	—	—	—	—	1.00 (0.98–1.02)	0.970
CS								
0	1		—	—	—	—	1	
1	4.28 (1.98–9.26)	**<0.001**	—	—	—	—	2.70 (1.22–5.96)	**0.014**
2	8.03 (3.59–17.95)	**<0.001**	—	—	—	—	4.09 (1.72–9.70)	**0.001**
3	8.35 (3.91–17.85)	**<0.001**	—	—	—	—	3.75 (1.66–8.46)	**0.002**
4	11.10 (5.20–23.70)	**<0.001**	—	—	—	—	4.62 (2.03–10.50)	**<0.001**
Sodium (mmol × L^-1^)	0.94 (0.92–0.96)	**<0.001**	—	—	0.99 (0.96–1.02)	0.490	—	—
Creatinine (mg × dl^-1^)	1.98 (1.40–2.79)	**0.001**	—	—	1.23 (0.86–1.76)	0.260	—	—
Platelets (per 10 G × L^-1^)	0.99 (0.97–1.00)	0.120	0.99 (0.97–1.01)	0.260	1.00 (0.98–1.01)	0.620	1.00 (0.98–1.02)	0.870
Haematocrit (g × dl^-1^)	0.94 (0.92–0.95)	**<0.001**	0.94 (0.92–0.95)	**<0.001**	0.97 (0.95–0.99)	**0.014**	0.98 (0.96–1.00)	0.090
Albumin (g × L^-1^)	0.94 (0.93–0.96)	**<0.001**	—	—	—	—	0.99 (0.97–1.00)	0.140
CRP (mg × dl^-1^)	1.71 (1.50–1.96)	**<0.001**	—	—	1.21 (1.01–1.44)	**0.037**	1.24 (1.04–1.48)	**0.016**
CT/PFA-100 (per 10 s)	1.02 (1.01–1.03)	**0.005**	1.00 (0.99–1.02)	0.970	1.00 (0.99–1.02)	0.690	1.01 (0.99–1.02)	0.520

Parameters include platelets and haematocrit (model 1); CTP score, serum sodium, and creatinine (model 2); or UNOS MELD (2016) score, CS, and serum albumin (model 3) with removal of the primary aetiological factor/requirement of liver transplantation/non-liver-related death as competing risks. Values of *p* in bold denote *p* <0.05. aSHR, adjusted SHR; CRP, C-reactive protein; CS, clinical stage; CT, closure time; CTP, Child–Turcotte–Pugh; HVPG, hepatic venous pressure gradient; PFA-100, Platelet Function Analyzer 100; PLT, platelet count; SHR, subdistribution hazard ratio; UNOS MELD (2016), United Network for Organ Sharing model for end-stage liver disease (2016).

**Table 3:** Univariable and multivariable competing risk regression analyses of factors associated with liver-related death.

Patient characteristics	Univariable		Model 1 (including PLT and haematocrit)		Model 2 (including CTP score, sodium, and creatinine)		Model 3 (including MELD, CS, and albumin)	
	SHR (95% CI)	*p* value	aSHR (95% CI)	*p* value	aSHR (95% CI)	*p* value	aSHR (95% CI)	*p* value
Age (year)	1.03 (1.02–1.05)	**<0.001**	—	—	1.03 (1.01–1.05)	**<0.001**	1.03 (1.01–1.05)	**<0.001**
HVPG (mmHg)	1.10 (1.07–1.12)	**<0.001**	—	—	1.05 (1.02–1.09)	**0.002**	1.04 (1.00–1.08)	**0.032**
CTP score								
A	1		—	—	1		—	—
B	2.36 (1.71–3.25)	**<0.001**	—	—	1.30 (0.87–1.95)	0.200	—	—
C	3.80 (2.48–5.82)	**<0.001**	—	—	1.75 (0.99–3.11)	0.056	—	—
UNOS MELD (2016) score (point)	1.07 (1.05–1.11)	**<0.001**	—	—	—	—	1.01 (0.97–1.04)	0.790
CS								
0	1		—	—	—	—	1	
1	8.40 (2.02–34.90)	**0.003**	—	—	—	—	4.35 (1.03–18.46)	**0.046**
2	16.00 (3.69–69.30)	**<0.001**	—	—	—	—	6.46 (1.41–29.69)	**0.016**
3	14.40 (3.48–59.90)	**<0.001**	—	—	—	—	4.74 (1.07–20.91)	**0.040**
4	21.30 (5.16–87.90)	**<0.001**	—	—	—	—	6.30 (1.42–28.01)	**0.016**
Sodium (mmol × L^-1^)	0.93 (0.90–0.97)	**<0.001**	—	—	0.99 (0.95–1.04)	0.770	—	—
Creatinine (mg × dl^-1^)	2.07 (1.26–3.41)	**0.004**	—	—	1.13 (0.67–1.91)	0.650	—	—
Platelets (per 10 G × L^-1^)	0.97 (0.95–0.99)	**0.045**	0.98 (0.96–1.01)	0.160	0.99 (0.96–1.02)	0.430	1.00 (0.97–1.02)	0.740
Haematocrit (g × dl^-1^)	0.93 (0.90–0.95)	**<0.001**	0.93 (0.91–0.96)	**<0.001**	0.97 (0.94–1.01)	0.130	0.98 (0.94–1.02)	0.300
Albumin (g × L^-1^)	0.93 (0.91–0.96)	**<0.001**	—	—	—	—	0.98 (0.95–1.01)	0.180
CRP (mg × dl^-1^)	1.87 (1.54–2.28)	**<0.001**	—	—	1.31 (0.99–1.72)	0.060	1.36 (1.03–1.79)	**0.033**
CT/PFA-100 (per 10 s)	1.03 (1.01–1.05)	**0.003**	1.01 (0.99–1.03)	0.520	1.01 (0.99–1.03)	0.350	1.01 (0.99–1.04)	0.250

Parameters include platelets and haematocrit (model 1); CTP score, sodium, and creatinine (model 2); or UNOS MELD (2016) score, CS, and serum albumin (model 3) with removal of the primary aetiological factor/requirement of liver transplantation/non-liver-related death as competing risks. Values of *p* in bold denote *p* <0.05. aSHR, adjusted SHR; CRP, C-reactive protein; CS, clinical stage; CT, closure time; CTP, Child–Turcotte–Pugh; HVPG, hepatic venous pressure gradient; PFA-100, Platelet Function Analyzer 100; PLT, platelet count; SHR, subdistribution hazard ratio; UNOS MELD (2016), United Network for Organ Sharing model for end-stage liver disease (2016).

**Fig. 3:**
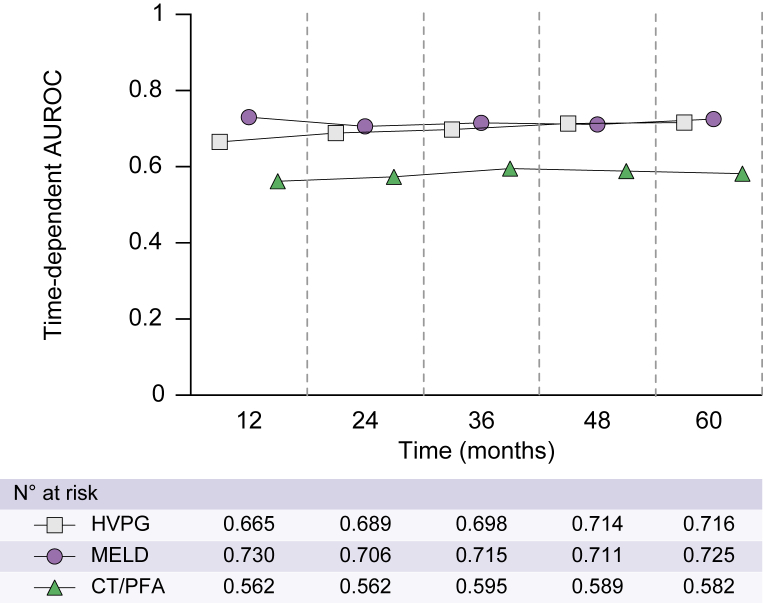
**CT/PFA-100 as a prognostic marker.** Comparing the time-dependent AUROCs for HVPG, UNOS MELD (2016), and CT/PFA-100 for hepatic decompensation/liver-related death. Time-dependent AUROCs for HVPG, UNOS MELD (2016), and CT/PFA-100 were calculated and plotted for the following time points: 12, 24, 36, 48, and 60 months. AUROC, area under the receiver operating characteristic curve; CT, closure time; HVPG, hepatic venous pressure gradient; PFA-100, Platelet Function Analyzer 100; UNOS MELD (2016), United Network for Organ Sharing model for end-stage liver disease (2016).

Next, we evaluated the prognostic performance of CT and compared it with UNOS MELD (2016) and HVPG in time-dependent AUROC analyses. Importantly, time-dependent AUROCs of CT for hepatic decompensation/liver-related death were clinically meaningless and inferior to those of the HVPG and UNOS MELD (2016) score at all tested time points (Fig. 3).

Finally, in patients with decompensated ACLD, the associations of CT and the Chronic Liver Failure Consortium ACLF-Development score (CLIF-C ACLF-D score) with the development of ACLF/liver-related death during follow-up were evaluated (Table S11). Although there was a trend in univariable analysis, PFA-100 CT was not associated with outcome in a multivariable analysis adjusted for the CLIF-C ACLF-D score.

### Associations of CT and coagulation events during follow-up

Table S12 depicts the prevalence of bleeding/thrombotic events in our cohort during the median follow-up of 62.4 (95% CI 55.7–69.0) months. Most importantly, PFA-100 CT was not associated with any major bleedings, major portal-hypertensive bleedings, or any non-malignant thromboses in univariable competing risk regression analyses (Table 4). Finally, not even the absence of platelet plug formation after the maximum observation time (*i.e*. 300 s) was associated with an increased risk of bleeding.

**Table 4:** Univariable competing risk regression analyses of bleeding/thrombotic events with initiation of anti-platelet therapy/anticoagulation/death as competing risks.

Patient characteristics	Any major bleeding (n = 92)		Major portal-hypertensive bleeding (n = 81)		Any non-malignant thrombosis (n = 79)	
	HR (95% CI)	*p* value	HR (95% CI)	*p* value	HR (95% CI)	*p* value
CT/PFA-100 (per 10 s)	1.01 (0.99–1.03)	0.340	1.01 (0.99–1.04)	0.330	0.99 (0.97–1.02)	0.580

CT, closure time; HR, hazard ratio; PFA-100, Platelet Function Analyzer 100.

## Discussion

In contrast to the historic concept of a platelet function defect in patients with ACLD, recent evidence indicates that platelets of patients with ACLD may be hyperactive upon stimulation with agonists.^2^ Intriguingly, the increased aggregation of individual platelets upon activation (as evaluated by the PLT ratio) has been linked to poor clinical outcomes in patients with decompensated cirrhosis.

The findings of our study on PFA-100 CT, that is, a test that has been referred to as ‘*in vitro* bleeding time’ and is performed under shear (*i.e*. more physiological) conditions, suggest that the primary haemostatic capacity is commonly impaired in patients with ACLD and decreases proportional to ACLD severity. However, there was no consistent independent association with decompensation/liver-related death or bleeding/thrombotic events in our large, thoroughly characterised cohort of patients with long-term follow-up.

Our study emphasises that the primary haemostatic capacity – assessed *in vitro* under shear stress by PFA-100 – was commonly impaired in patients with ACLD and decreased proportional to ACLD severity, which is likely related to accompanying decreases in PLT and haematocrit,^6^ which were major determinants of CT in our study. Notably, patients with antiplatelet/anticoagulation therapy at baseline were excluded, and thus, these medications did not impact our results. Interestingly, platelet adhesion was supported by plasma containing high VWF levels in a study by Lisman *et al.*^16^ However, we observed no independent association between VWF and CT (Table S13), which could be explained by a ceiling effect in patients with ACLD who have universally high VWF, although such a ceiling effect was not evident in a previous study on PFA-100 in patients with cirrhosis.^17^ However, VWF ristocetin co-factor (available in 79% of patients) – reflecting functional activity of VWF within plasma – seemed to be a determinant of PFA-100 CT (Table S14). Notably, we abstained from adjusting for VWF-Ag in further analyses and rather focused on PLT and haematocrit, which emerged as the main determinants of PFA-100 CT.

Results from animal models support the concept that alterations in platelet function promote liver disease progression.^18^ Based on this hypothesis, Zanetto *et al.*^3^ demonstrated in a comparatively small cohort of patients with decompensated cirrhosis that whole blood platelet aggregation is significantly increased when accounting for thrombocytopaenia. For this purpose, a ratio of platelet aggregation and PLT was calculated, and high PLT ratio values identified patients with poor short-term outcomes (increased risks of further hepatic decompensation and death).^3^ Importantly, the authors did not adjust for the severity of systemic inflammation, a well-known driver of first and further hepatic decompensation and mortality,[19], [20], [21] which may have been a confounder, as the underlying bacterial translocation increases PLT activatability by agonists.^2^ Accordingly, whether the increased aggregation of individual PLT upon activation by agonists is independently associated and even drives the progression of liver disease or simply is a biomarker of systemic inflammation or other accompanying pathophysiological mechanisms remains unanswered.^22^ As patients with evidence of bacterial infections were excluded from our study, median CRP levels were low. Even ‘sophisticated’ biomarkers of bacterial translocation/systemic inflammation had no influence on PFA-100/CT in fully adjusted models. It is unclear whether this also applies to less stable patients and might be generalisable. Our findings do not support a direct biological relevance, as its downstream consequence – a more active primary haemostatic system – was not linked to adverse clinical outcomes. Although a preserved primary haemostatic capacity was even linked to better outcomes in univariable analysis, after accounting for PLT and haematocrit as determinants of CT beyond platelet function and/or well-established prognostic indicators including systemic inflammation in multivariable analysis, no association was observed. Alternatively, when correcting the CT for thrombocytopaenia and anaemia using a previously published formula considering PLT/haematocrit,^5^ the corrected CT showed some association with adverse clinical outcomes; however, in contrast to what would have been expected by the data of Zanetto *et a*.,^3^ higher corrected CT (*i.e.* longer time to platelet plug formation) was associated with an increased risk of hepatic decompensation/liver-related death. In addition, no independent associations with liver-related death in the overall cohort or ACLF/liver-related death in our large sample of decompensated patients were observed.

Notably, there are several important methodological differences (*e.g.* the presence/absence of shear stress) between Multiplate and PFA-100, which prevent direct comparisons and may explain discrepancies in findings. They explore different parts/aspects of haemostasis and provide different types of results/information. Moreover, in the prospectively collected cohort of patients with cirrhosis tested by Multiplate,^3^ patients were considerably older, and more than one-third of patients were patients with CTP C (*vs*. 12% in our study). Although severity of systemic inflammation (*i.e.* CRP levels) was not reported, 30% of patients were (suspected to be) infected. In contrast, we excluded patients with bacterial infection, and median CRP levels were low (*i.e.* 0.3 [IQR 0.1–0.7] mg × L^-1^). Although we understand that data from consecutively included ‘real-life’ patients are valuable, bacterial infections or bursts of sterile inflammation might hinder firm conclusions, especially when the main results are not adjusted for a well-established disease-driving mechanisms (*e.g.* systemic inflammation^23^).

Although changes in primary haemostasis have been implicated in bleeding and thrombosis in patients with ACLD, CT/PFA-100 was not associated with coagulation-related events in our study. Notably, a previous study reported a (VWF-independent) link between CT/PFA-100 and venous thromboembolism, while not observing such an association for Multiplate results. This suggests that in general, CT/PFA-100 is capable of detecting prothrombotic changes. Using a similar methodology as in their previously mentioned study, Zanetto *et al.*^24^ showed that PLT ratio was also associated with PVT development in patients with decompensated cirrhosis. The authors hypothesised that a combination of altered endothelium and activated platelets contribute to the development of PVT in cirrhosis,^13^ although similar concerns regarding the interpretation of the PLT ratio may apply. Moreover, it is worth mentioning that a hyperactive coagulation system has also been implicated in the pathophysiology of PVT in cirrhosis; however, a recent study questioned the postulated differences in laboratory tests of coagulation between the portal venous and systemic circulation,^25^ and Turon *et al.*^26^ found that portal hypertension severity rather than laboratory tests of coagulation predicts PVT development. Finally, accumulating evidence suggests that laboratory studies only scratch the surface of the pathophysiology of thrombosis in ACLD.^27^ In this context, Driever *et al.*^28^ demonstrated that portal vein ‘thrombosis’ is merely a result of intimal thickening compared with a truly ‘thrombotic’ problem, which question the role of haemostasis/coagulation in the development of PVT (*i.e.* the most common thrombotic event in patients with ACLD). However, patients with ACLD and PVT who receive anticoagulant therapy have increased recanalisation and reduced progression rates, as compared with patients who do not receive anticoagulants,^29^ arguing for the importance of the haemostatic system.

The main limitation of our study is its retrospective design. However, patients included in our study were thoroughly characterised in terms of portal hypertension and systemic inflammation severity, prognostic scores, and routine laboratory parameters including PLT/haematocrit values. Importantly, all of these aspects have been considered in our analyses, thereby limiting the possibility of unaccounted confounding. Furthermore, we cannot exclude that some hepatic decompensation events have been missed. However, we have thoroughly reviewed electronic health records of the Vienna hospital association and nationwide electronic health records. Moreover, we have also performed searches of the liver transplant database of our institution (*i.e.* the only transplant centre in eastern Austria) and examined the nationwide death registry. As complete information on (reason of) death is guaranteed by the latter measure, we included liver-related death in all composite endpoints to ensure the ascertainment of the most severe disease courses. We cannot rule out selection bias, as we only included patients undergoing HVPG measurement with information on PFA-100. However, haemodynamic evaluations are routinely performed for risk stratification and treatment monitoring purposes at our centre, and thus, we are confident that our study population is quite representative. Moreover, the availability of information on HVPG is a strength of our study, as it allows us to adjust for the severity of portal hypertension as evaluated by the minimally invasive gold-standard test;^9^ however, information on its prognostic utility should not be overinterpreted, as HVPG measurements were performed in the absence of non-selective betablockers, which may have modified both HVPG and risk during follow-up.^30^ Furthermore, a considerable number of patients had to be excluded owing to missing information on PFA-100, which is explained by the fact that laboratory workup in patients undergoing HVPG measurement was not constant over time. Nevertheless, our study comprises by far the largest ACLD cohort that has been evaluated by PFA-100, and the absence of other reasonably sized cohorts in the literature/requirement of fresh samples explains why our findings on clinical outcomes have not been validated. Finally, PFA-100 testing has inherent limitations (*e.g*. it does not cover all aspects of primary haemostasis), and – as with all other laboratory tests for platelet function/primary haemostasis – the biological relevance on *in vitro* findings remains unclear.

In conclusion, our study suggests that the primary haemostatic capacity – assessed *in vitro* under shear stress by PFA-100 – was commonly impaired in patients with ACLD and decreased proportional to ACLD severity. Intriguingly, higher CT values even tended to be independently associated with an increased risk of decompensation/liver-related death or bleeding/thrombotic events in our large, thoroughly characterised cohort with long-term follow-up. These findings do not support the hypothesis that increased platelet adhesion – assessed *in vitro* under shear stress by PFA-100 – promotes ACLD progression.

## Financial support

No financial support specific to this study was received.

## Authors’ contributions

Concept of the study: LB, MM. Data collection: LB, BSc, RP, BSi, LH, MJ, GS, BSH, AFS, MP, PQ, TR, MM. Statistical analyses: LB, MM. Drafting of the manuscript: LB, MM. Revision for important intellectual content and approval of the final manuscript: all authors.

## Data availability statement

The data that support the findings of this study are available from the corresponding author upon reasonable request.

## Conflicts of interest

RP received travel support from AbbVie, Gilead, and Takeda. BSc received travel support from AbbVie, Ipsen, and Gilead. BSi received travel support from AbbVie and Gilead. MP served as a speaker and/or consultant and/or advisory board member for Bayer, Bristol-Myers Squibb, Eisai, Ipsen, Lilly, MSD, and Roche and received travel support from Bayer and Bristol-Myers Squibb. MT served as a speaker and/or consultant and/or advisory board member for Albireo, BiomX, Boehringer Ingelheim, Bristol-Myers Squibb, Falk, Genfit, Gilead, Intercept, Janssen, MSD, Novartis, Phenex, Pliant, Regulus, and Shire and received travel support from AbbVie, Falk, Gilead, and Intercept as well as grants/research support from Albireo, Alnylam, Cymabay, Falk, Gilead, Intercept, MSD, Takeda, and UltraGenyx. He is also co-inventor of patents on the medical use of 24-norursodeoxycholic acid. TR served as a speaker and/or consultant and/or advisory board member for and received speaking honoraria from AbbVie, Bayer, Boehringer-Ingelheim, Gilead, Intercept, MSD, Roche, Siemens, and W. L. Gore & Associates and received travel support from AbbVie, Boehringer-Ingelheim, Gilead, and Roche as well as grants/research support from AbbVie, Boehringer-Ingelheim, Gilead, Intercept, MSD, Myr Pharmaceuticals, Philips Healthcare, Pliant, Siemens, and W. L. Gore & Associates. MM served as a speaker and/or consultant and/or advisory board member for AbbVie, Collective Acumen, Echosens, Gilead, Takeda, and W. L. Gore & Associates and received travel support from AbbVie and Gilead. All other authors declared no conflicts of interest.

Please refer to the accompanying ICMJE disclosure forms for further details.
