# Supplementary material for: Platelet adhesion assessed by PFA-100 is not linked to progression of ACLD
Source: JHEP Rep. 2023 Oct 12;6(1):100934. doi: 10.1016/j.jhepr.2023.100934 (PMC10698528; doi:10.1016/j.jhepr.2023.100934)
Supplement: Multimedia component 1 [file mmc1.pdf]

Platelet adhesion assessed by PFA-100 is not linked to progression of ACLD

Lorenz Balcar, Benedikt Simbrunner, Rafael Paternostro, Mathias Jachs, Lukas Hartl, Georg Semmler, Benedikt Silvester Hofer, Albert Friedrich Stättermayer, Matthias Pinter, Michael Trauner, Peter Quehenberger, Ton Lisman, Thomas Reiberger, Bernhard Scheiner, Mattias Mandorfer

Table of contents

Table S1 2
Table S2 4
Table S3 5
Table S4 6
Table S5 7
Table S6 9
Table S7 11
Table S8 13
Table S9 15
Table S10 17
Table S11 19
Table S12 20
Table S13 21
Table S14 23
Supplementary reference .... **Error! Bookmark not defined.**

Supplementary tables

Table S1

Patient characteristics	**Study cohort, n=688**
Age, years, mean ± SD	53.2±11.3
Body mass index, kg x m^{-2}	26.2±5.3
Sex, n (%)	
Male	459 (67%)
Female	229 (33%)
Aetiology, n (%)	
ArLD	281 (41%)
Viral	259 (38%)
NAFLD	53 (8%)
Other	95 (14%)
Varices, n (%) *	434 (70%)
History of variceal bleeding, n (%)	124 (18%)
Decompensated, n (%)	398 (58%)
Stages of decompensation, n (%)	
Stable decompensated cirrhosis	328 (48%)
Unstable decompensated cirrhosis	55 (8%)
Pre-acute-on-chronic liver failure (ACLF)	15 (2%)
Ascites, n (%)	
None	379 (55%)
Mild	236 (34%)
Severe	73 (11%)
History of/current overt hepatic encephalopathy, n (%)	148 (21%)
HVPG, mmHg, mean ± SD	17±6
HVPG 6-9 mmHg, n (%)	89 (13%)
HVPG 10-15 mmHg, n (%)	178 (26%)
HVPG ≥16 mmHg, n (%)	421 (61%)
UNOS MELD (2016), points, mean ± SD	13±5
CTP score, points, mean ± SD	7±2
A, n (%)	357 (52%)
B, n (%)	253 (37%)
C, n (%)	78 (11%)
Clinical stages, n (%)	
CS 0	76 (11%)
CS 1	214 (31%)
CS 2	45 (7%)
CS 3	193 (28%)
CS 4	160 (23%)
NSBB exposure during follow-up	

No initiation/never	240 (35%)
Minority of time	95 (14%)
Majority of time	110 (16%)
All of the time	243 (35%)
Laboratory parameters, median (IQR) or mean ± SD	
Mean arterial pressure, mmHg	98±14
Platelet count, G x L^{-1}	102 (69-142)
Haematocrit, %	34.1±5.7
Haemoglobin, g x dL^{-1}	11.8±2.1
PFA-100, s	180 (135-300)
Sodium, mmol x L^{-1}	137.3±4.2
Albumin, g x L^{-1}	35.2±5.8
Bilirubin, mg x dL^{-1}	1.2 (0.8-2.2)
INR, %	1.4±0.3
Creatinine, mg x dL^{-1}	0.8 (0.6-0.9)
von Willebrand factor-antigen	304 (232-398)
CRP, mg x dL^{-1}	0.3 (0.1-0.7)

Categorical variables were reported as absolute (n) and relative frequencies (%), whereas continuous variables as mean ± SD or median (interquartile range [IQR]), as appropriate.

Table S1. Detailed patient characteristics at the time of HVPG measurement.

Abbreviations: ARLD alcohol-related liver disease; CRP C-reactive protein; CS clinical stage; CTP Child-Turcotte-Pugh; HVPG hepatic venous pressure gradient; INR international normalized ratio; NAFLD non-alcoholic fatty liver disease; NSBB non-selective betablocker; PFA-100 Platelet Function Analyzer 100; UNOS MELD (2016) score United Network for Organ Sharing model for end-stage liver disease (2016)

Table S2

Disease severity indices	**Number of patients, n (%)**	**CT/PFA-100, median (IQR)**	**p-value**
CTP score			
A	357 (52%)	167 (135-300)	**0.009**
B	253 (37%)	194 (133-300)	
C	78 (11%)	250 (153-300)	
UNOS MELD (2016) score			
<10	228 (33%)	160 (130-279)	**<0.001**
10-14	258 (37%)	197 (144-300)	
≥15	202 (29%)	203 (138-300)	
HVPG, mmHg			
HVPG 6-9 mmHg	89 (13%)	145 (116-198)	**0.002**
HVPG 10-15 mmHg	178 (26%)	160 (130-300)	
HVPG ≥16 mmHg	421 (61%)	202 (146-300)	
Clinical Stages			
CS 0	76 (11%)	149 (120-200)	**<0.001**
CS 1	214 (31%)	184 (139-300)	
CS 2	45 (7%)	198 (148-300)	
CS 3	193 (28%)	167 (131-300)	
CS 4	160 (23%)	226 (143-300)	
History of decompensation			
cACLD	290 (42%)	170 (134-300)	0.075
dACLD	398 (58%)	192 (135-300)	
Stages of decompensation			
SDC	328 (48%)	185 (135-300)	0.202
UDC	55 (8%)	210 (145-300)	
pre-ACLF	15 (2%)	300 (141-400)	

CT/PFA-100 values were displayed as median (interquartile range [IQR]). Mann-Whitney-U-test was used for group comparisons.

P-values in bold denote p<0.05.

Table S2. Comparison of CT/PFA-100 according to different disease severity indices.

Abbreviations: c/dACLD compensated/decompensated advanced chronic liver disease; CS clinical stage; CT clotting time; CTP Child-Turcotte-Pugh score; HVPG hepatic venous pressure gradient; PFA-100 Platelet Function Analyzer 100; (pre)-ACLF pre-acute-on-chronic liver failure; SDC stable decompensated cirrhosis; UDC unstable decompensated cirrhosis; UNOS MELD (2016) score United Network for Organ Sharing model for end-stage liver disease (2016) score

Table S3

Disease severity indices	**Number of patients, n (%)**	**PLT, median (IQR)**	**p-value**
CTP score			
A	357 (52%)	104 (72-145)	0.087
B	253 (37%)	104 (71-142)	
C	78 (11%)	83 (61-124)	
UNOS MELD (2016) score			
<10	228 (33%)	115 (86-164)	**<0.001**
10-14	258 (37%)	92 (63-127)	
≥15	202 (29%)	99 (65-139)	
HVPG, mmHg			
HVPG 6-9 mmHg	89 (13%)	151 (106-189)	**0.019**
HVPG 10-15 mmHg	178 (26%)	106 (78-156)	
HVPG ≥16 mmHg	421 (61%)	94 (64-124)	
Clinical Stages			
CS 0	76 (11%)	152 (109-180)	**<0.001**
CS 1	214 (31%)	96 (66-124)	
CS 2	45 (7%)	84 (54-110)	
CS 3	193 (28%)	106 (80-151)	
CS 4	160 (23%)	94 (60-128)	
History of decompensation			
cACLD	290 (42%)	105 (72-147)	0.120
dACLD	398 (58%)	100 (67-137)	
Stages of decompensation			
SDC	328 (48%)	100 (67-136)	0.962
UDC	55 (8%)	106 (68-142)	
pre-ACLF	15 (2%)	107 (59-137)	

PLT values were displayed as median (interquartile range [IQR]). Mann-Whitney-U-test was used for group comparisons. P-values in bold denote $p<0.05$.

Table S3. Comparison of platelet counts according to different disease severity indices.

Abbreviations: c/dACLD compensated/decompensated advanced chronic liver disease; CS clinical stage; CTP Child-Turcotte-Pugh score; HVPG hepatic venous pressure gradient; PLT platelets; (pre)-ACLF pre-acute-on-chronic liver failure; SDC stable decompensated cirrhosis; UDC unstable decompensated cirrhosis; UNOS MELD (2016) score United Network for Organ Sharing model for end-stage liver disease (2016) score

Table S4

Disease severity indices	**Number of patients, n (%)**	**Haematocrit, mean ± SD**	**p-value**
CTP score			
A	357 (52%)	36.5±5.2	**<0.001**
B	253 (37%)	31.8±5.2	
C	78 (11%)	30.3±4.9	
UNOS MELD (2016) score			
<10	228 (33%)	36.6±5.5	**<0.001**
10-14	258 (37%)	34.0±5.3	
≥15	202 (29%)	31.3±5.3	
HVPG, mmHg			
HVPG 6-9 mmHg	89 (13%)	38.2±5.3	**<0.001**
HVPG 10-15 mmHg	178 (26%)	35.5±5.4	
HVPG ≥16 mmHg	421 (61%)	32.6±5.3	
Clinical Stages			
CS 0	76 (11%)	38.6±5.2	**<0.001**
CS 1	214 (31%)	36.5±5.0	
CS 2	45 (7%)	31.7±5.4	
CS 3	193 (28%)	32.6±5.4	
CS 4	160 (23%)	31.1±4.6	
History of decompensation			
cACLD	290 (42%)	37.1±5.1	**<0.001**
dACLD	398 (58%)	32.0±5.1	
Stages of decompensation			
SDC	328 (48%)	32.3±5.1	**0.003**
UDC	55 (8%)	30.1±4.4	
pre-ACLF	15 (2%)	29.6±7.5	

Haematocrit values were displayed as mean ± SD. Student's t-test was used for group comparisons. P-values in bold denote $p<0.05$.

Table S4. Comparison of haematocrit values according to different disease severity indices.

Abbreviations: c/dACLD compensated/decompensated advanced chronic liver disease; CS clinical stage; CTP Child-Turcotte-Pugh score; HVPG hepatic venous pressure gradient; UNOS MELD (2016) score United Network for Organ Sharing model for end-stage liver disease (2016) score

Table S5

Patient characteristics	**'Sophisticated' SI-biomarker available, n=202 (29%)**	**'Sophisticated' SI-biomarker not available, n=486 (71%)**	**p-value**
Age, years, mean ± SD	53.7±12.1	52.9±11.0	0.401
Body mass index, kg x m^{-2}	26.4±5.2	26.1±5.4	0.482
Sex, n (%)			
Male	133 (66%)	326 (67%)	0.754
Female	69 (34%)	160 (33%)	
Aetiology, n (%)			
ArLD	99 (49%)	182 (37%)	**<0.001**
Viral	43 (21%)	216 (44%)	
NAFLD	18 (9%)	35 (7%)	
Other	42 (21%)	53 (11%)	
Varices, n (%) *	127 (65%)	207 (73%)	**<0.001**
History of variceal bleeding, n (%)	28 (14%)	96 (20%)	0.067
Decompensated, n (%)	132 (65%)	266 (55%)	**0.010**
Stages of decompensation, n (%)			
Stable decompensated cirrhosis	104 (52%)	224 (46%)	**0.030**
Unstable decompensated cirrhosis	21 (10%)	34 (7%)	
Pre-acute-on-chronic liver failure (ACLF)	7 (4%)	8 (2%)	
Ascites, n (%)			
None	90 (45%)	289 (60%)	**<0.001**
Mild	94 (47%)	142 (29%)	
Severe	18 (9%)	55 (11%)	
History of/current overt hepatic encephalopathy, n (%)	42 (21%)	106 (22%)	0.076
HVPG, mmHg, mean ± SD	17±6	17±6	0.472
HVPG 6-9 mmHg, n (%)	18 (9%)	71 (15%)	0.115
HVPG 10-15 mmHg, n (%)	57 (28%)	121 (25%)	
HVPG ≥16 mmHg, n (%)	127 (63%)	294 (61%)	
UNOS MELD (2016), points, mean ± SD	13±5	12±5	0.052
CTP score, points, mean ± SD	7±2	7±2	0.460
A, n (%)	95 (47%)	262 (54%)	0.054
B, n (%)	88 (44%)	165 (34%)	
C, n (%)	19 (9%)	59 (12%)	
Clinical stages, n (%)			
CS 0	15 (7%)	61 (13%)	**<0.001**
CS 1	55 (27%)	159 (33%)	
CS 2	8 (4%)	37 (8%)	

CS 3	81 (40%)	112 (23%)	
CS 4	43 (21%)	117 (24%)	
Laboratory parameters, median (IQR) or mean ± SD			
Mean arterial pressure, mmHg	97±15	99±14	0.139
Platelet count, G x L^{-1}	100 (69-138)	104 (69-143)	0.609
Haematocrit, %	33.1±5.7	34.5±5.7	**0.005**
Haemoglobin, g x dL^{-1}	11.4±2.1	11.9±2.1	**0.007**
PFA-100, s	164 (135-300)	185 (135-300)	0.608
Sodium, mmol x L^{-1}	137.8±3.9	137.1±4.3	0.063
Albumin, g x L^{-1}	35.4±5.2	35.2±6.1	0.658
Bilirubin, mg x dL^{-1}	1.2 (0.8-2.1)	1.2 (0.8-2.2)	0.952
INR, %	1.5±0.3	1.3±0.3	**<0.001**
Creatinine, mg x dL^{-1}	0.7 (0.6-0.9)	0.8 (0.7-0.9)	**0.022**
von Willebrand factor-antigen	287 (231-371)	314 (232-403)	**0.029**
CRP, mg x dL^{-1}	0.3 (0.1-0.6)	0.3 (0.1-0.7)	0.853

Table S5. Comparison of patients with vs. without available data on 'sophisticated' biomarkers of bacterial translocation/systemic inflammation.

Abbreviations: ARLD alcohol-related liver disease; CRP C-reactive protein; CS clinical stage; CTP Child-Turcotte-Pugh; HVPG hepatic venous pressure gradient; INR international normalized ratio; NAFLD non-alcoholic fatty liver disease; NSBB non-selective betablocker; PFA-100 Platelet Function Analyzer 100; UNOS MELD (2016) score United Network for Organ Sharing model for end-stage liver disease (2016)

Table S6

Patient characteristics available in n=202 (29%) of patients	**Univariable**		**Model 1 (incl. CTP score, sodium, and creatinine)**		**Model 2 (incl. MELD, CS, and albumin)**	
	**B**	**p-value**	**B**	**p-value**	**B**	**p-value**
Age, year	1.493	**0.001**	1.178	**0.008**	1.312	**0.002**
Male sex	-2.306	0.849	-	-	-	-
BMI, kg x m^{-2}	1.102	0.319	-	-	-	-
Overweight[1]	13.987	0.228	-	-	-	-
Obesity[2]	17.692	0.172	-	-	-	-
Prediabetes[3]	0.675	0.070	0.942	**0.004**	0.837	**0.011**
Diabetes[4]	1.286	0.930	-	-	-	-
Arterial hypertension[5]	1.089	0.930	-	-	-	-
Hypertriglyceridemia[6]	-34.887	0.169	-	-	-	-
Hypercholesterolemia[7]	-19.370	0.295	-	-	-	-
HDL below threshold[8]	7.991	0.529	-	-	-	-
Statin use	-33.474	0.285	-	-	-	-
Hepatic steatosis[9]	10.310	0.410	-	-	-	-
CTP score, point	10.293	**0.001**	6.806	0.060	-	-
UNOS MELD (2016), point	3.343	**0.007**	-	-	1.585	0.253
HVPG, mmHg	2.059	**0.040**	-0.498	0.630	-0.244	0.813
dACLD (CS 2-4)	6.523	0.589	-	-	-18.649	0.125
Platelet count, G x L^{-1}	-0.414	**<0.001**	-0.430	**<0.001**	-0.444	**<0.001**
Haematocrit, %	-4.538	**<0.001**	-4.299	**<0.001**	-4.905	**<0.001**
Sodium, mmol x L^{-1}	0.550	0.713	1.475	0.356	-	-
Creatinine, mg x dL^{-1}	31.523	0.110	15.799	0.386	-	-
Albumin, g x L^{-1}	-2.758	**0.013**	-	-	-0.543	0.648
LBP, ng x mL^{-1}	0.165	0.823	0.325	0.615	0.359	0.581

[1]BMI ≥25 kg x m^{-2}
[2]BMI ≥30 kg x m^{-2}
[3]Fasting blood glucose 100-125mg x dL^{-1}; HbA1c 5.7-6.4%
[4]Fasting blood glucose >125mg x dL^{-1}, HbA1c ≥6.5%, or antidiabetic medication
[5]Blood pressure >140/90mmHg, or antihypertensive medication
[6]Triglycerides >150 mg x dL^{-1}
[7]Total cholesterol >200 mg x dL^{-1}
[8]<35mg x dL^{-1} for males and <39mg x dL^{-1} for females
[9]Biopsy-proven, controlled attenuation parameter >248dB x m^{-1}, or diagnosed by ultrasound
P-values in bold denote p<0.05.

Table S6. Simple and multiple linear regression analysis of factors associated with CT/PFA-100 including – among other parameters – either CTP score, sodium, creatinine, and lipopolysaccharide-binding protein **(model 1)**, or UNOS MELD (2016) score, CS, albumin, and lipopolysaccharide-binding protein **(model 2)**.

Abbreviations: ACLF acute-on-chronic liver failure; BMI body mass index; CRP C-reactive protein; CS clinical stage; CT clotting time; CTP Child-Turcotte-Pugh score; HVPG hepatic venous pressure gradient; LBP lipopolysaccharide-binding protein; PFA-100 Platelet Function Analyzer 100; UNOS MELD (2016) score United Network for Organ Sharing model for end-stage liver disease (2016) score

Table S7

Patient characteristics available in n=202 (29%) of patients	**Univariable**		**Model 1 (incl. CTP score, sodium, and creatinine)**		**Model 2 (incl. MELD, CS, and albumin)**	
	**B**	**p-value**	**B**	**p-value**	**B**	**p-value**
Age, year	1.493	**0.001**	1.186	**0.008**	1.318	**0.002**
Male sex	-2.306	0.849	-	-	-	-
BMI, kg x m^{-2}	1.102	0.319	-	-	-	-
Overweight[1]	13.987	0.228	-	-	-	-
Obesity[2]	17.692	0.172	-	-	-	-
Prediabetes[3]	0.675	0.070	0.934	**0.004**	0.829	**0.012**
Diabetes[4]	1.286	0.930	-	-	-	-
Arterial hypertension[5]	1.089	0.930	-	-	-	-
Hypertriglyceridemia[6]	-34.887	0.169	-	-	-	-
Hypercholesterolemia[7]	-19.370	0.295	-	-	-	-
HDL below threshold[8]	7.991	0.529	-	-	-	-
Statin use	-33.474	0.285	-	-	-	-
Hepatic steatosis[9]	10.310	0.410	-	-	-	-
CTP score, point	10.293	**0.001**	6.842	0.062	-	-
UNOS MELD (2016), point	3.343	**0.007**	-	-	1.542	0.270
HVPG, mmHg	2.059	**0.040**	-0.576	0.577	-0.336	0.743
dACLD (CS 2-4)	6.523	0.589	-	-	-18.424	0.129
Platelet count, G x L^{-1}	-0.414	**<0.001**	-0.431	**<0.001**	-0.449	**<0.001**
Haematocrit, %	-4.538	**<0.001**	-4.320	**<0.001**	-4.923	**<0.001**
Sodium, mmol x L^{-1}	0.550	0.713	1.404	0.382	-	-
Creatinine, mg x dL^{-1}	31.523	0.110	14.986	0.421	-	-
Albumin, g x L^{-1}	-2.758	**0.013**	-	-	-0.550	0.644
IL-6, pg x mL^{-1}	-0.005	0.977	0.032	0.831	0.075	0.614

[1]BMI ≥25 kg x m^{-2}
[2]BMI ≥30 kg x m^{-2}
[3]Fasting blood glucose 100-125mg x dL^{-1}; HbA1c 5.7-6.4%
[4]Fasting blood glucose >125mg x dL^{-1}, HbA1c ≥6.5%, or antidiabetic medication
[5]Blood pressure >140/90mmHg, or antihypertensive medication
[6]Triglycerides >150 mg x dL^{-1}
[7]Total cholesterol >200 mg x dL^{-1}
[8]<35mg x dL^{-1} for males and <39mg x dL^{-1} for females
[9]Biopsy-proven, controlled attenuation parameter >248dB x m^{-1}, or diagnosed by ultrasound
P-values in bold denote p<0.05.

Table S7. Simple and multiple linear regression analysis of factors associated with CT/PFA-100 including – among other parameters – either CTP score, sodium, creatinine, and interleukin-6 **(model 1)**, or UNOS MELD (2016) score, CS, albumin, and interleukin-6 **(model 2)**.

Abbreviations: ACLF acute-on-chronic liver failure; BMI body mass index; CRP C-reactive protein; CS clinical stage; CT clotting time; CTP Child-Turcotte-Pugh score; HVPG hepatic venous pressure gradient; IL-6 interleukine-6; PFA-100 Platelet Function Analyzer 100; UNOS MELD (2016) score United Network for Organ Sharing model for end-stage liver disease (2016) score

Table S8

Patient characteristics available in n=202 (29%) of patients	**Univariable**		**Model 1 (incl. CTP score, sodium, and creatinine)**		**Model 2 (incl. MELD, CS, and albumin)**	
	**B**	**p-value**	**B**	**p-value**	**B**	**p-value**
Age, year	1.493	**0.001**	1.175	**0.008**	1.292	**0.002**
Male sex	-2.306	0.849	-	-	-	-
BMI, kg x m^{-2}	1.102	0.319	-	-	-	-
Overweight[1]	13.987	0.228	-	-	-	-
Obesity[2]	17.692	0.172	-	-	-	-
Prediabetes[3]	0.675	0.070	0.925	**0.005**	0.827	**0.012**
Diabetes[4]	1.286	0.930	-	-	-	-
Arterial hypertension[5]	1.089	0.930	-	-	-	-
Hypertriglyceridemia[6]	-34.887	0.169	-	-	-	-
Hypercholesterolemia[7]	-19.370	0.295	-	-	-	-
HDL below threshold[8]	7.991	0.529	-	-	-	-
Statin use	-33.474	0.285	-	-	-	-
Hepatic steatosis[9]	10.310	0.410	-	-	-	-
CTP score, point	10.293	**0.001**	7.349	**0.041**	-	-
UNOS MELD (2016), point	3.343	**0.007**	-	-	1.708	0.216
HVPG, mmHg	2.059	**0.040**	-0.671	0.514	-0.413	0.687
dACLD (CS 2-4)	6.523	0.589	-	-	-16.422	0.181
Platelet count, G x L^{-1}	-0.414	**<0.001**	-0.433	**<0.001**	-0.433	**<0.001**
Haematocrit, %	-4.538	**<0.001**	-4.187	**<0.001**	-4.789	**<0.001**
Sodium, mmol x L^{-1}	0.550	0.713	1.259	0.431	-	-
Creatinine, mg x dL^{-1}	31.523	0.110	14.398	0.429	-	-
Albumin, g x L^{-1}	-2.758	**0.013**	-	-	-0.625	0.598
PCT, µg x L^{-1}	-3.106	0.178	-2.410	0.230	-2.049	0.310

[1]BMI ≥25 kg x m^{-2}
[2]BMI ≥30 kg x m^{-2}
[3]Fasting blood glucose 100-125mg x dL^{-1}; HbA1c 5.7-6.4%
[4]Fasting blood glucose >125mg x dL^{-1}, HbA1c ≥6.5%, or antidiabetic medication
[5]Blood pressure >140/90mmHg, or antihypertensive medication
[6]Triglycerides >150 mg x dL^{-1}
[7]Total cholesterol >200 mg x dL^{-1}
[8]<35mg x dL^{-1} for males and <39mg x dL^{-1} for females
[9]Biopsy-proven, controlled attenuation parameter >248dB x m^{-1}, or diagnosed by ultrasound
P-values in bold denote p<0.05.

Table S8. Simple and multiple linear regression analysis of factors associated with CT/PFA-100 including – among other parameters – either CTP score, sodium, creatinine, and procalcitonin **(model 1)**, or UNOS MELD (2016) score, CS, albumin, and procalcitonin **(model 2)**.

Abbreviations: ACLF acute-on-chronic liver failure; BMI body mass index; CRP C-reactive protein; CS clinical stage; CT clotting time; CTP Child-Turcotte-Pugh score; HVPG hepatic venous pressure gradient; PCT procalcitonin; PFA-100 Platelet Function Analyzer 100; UNOS MELD (2016) score United Network for Organ Sharing model for end-stage liver disease (2016) score

Table S9

Patient characteristics	**Univariable**		**Model 1** (incl. CTP score, sodium, and creatinine)		**Model 2** (incl. MELD, CS, and albumin)	
	**SHR (95%CI)**	**p-value**	**aSHR (95%CI)**	**p-value**	**aSHR (95%CI)**	**p-value**
Age, year	1.02 (1.01-1.03)	**<0.001**	1.02 (1.01-1.03)	**0.003**	1.02 (1.01-1.03)	**0.002**
HVPG, mmHg	1.08 (1.06-1.10)	**<0.001**	1.04 (1.02-1.07)	**<0.001**	1.03 (1.01-1.05)	**0.007**
CTP score						
A	1		1		-	-
B	2.34 (1.88-2.91)	**<0.001**	1.48 (1.13-1.93)	**0.004**	-	-
C	3.09 (2.30-4.15)	**<0.001**	1.66 (1.13-2.44)	**0.010**	-	-
UNOS MELD (2016) score, point	1.06 (1.04-1.08)	**<0.001**	-	-	1.00 (0.98-1.03)	0.870
CS						
CS 0	1		-	-	1	
CS 1	4.28 (1.98-9.26)	**<0.001**	-	-	2.68 (1.21-5.90)	**0.015**
CS 2	8.03 (3.59-17.95)	**<0.001**	-	-	4.23 (1.80-9.97)	**<0.001**
CS 3	8.35 (3.91-17.85)	**<0.001**	-	-	3.93 (1.75-8.85)	**<0.001**
CS 4	11.10 (5.20-23.70)	**<0.001**	-	-	4.83 (2.13-10.94)	**<0.001**
Sodium, mmol x L^{-1}	0.94 (0.92-0.96)	**<0.001**	0.99 (0.96-1.01)	0.310	-	-
Creatinine, mg x dL^{-1}	1.98 (1.40-2.79)	**0.001**	1.26 (0.88-1.81)	0.210	-	-
Albumin, g x L^{-1}	0.94 (0.93-0.96)	**<0.001**	-	-	0.99 (0.97-1.00)	0.130
CRP, mg x L^{-1}	1.71 (1.50-1.96)	**<0.001**	1.25 (1.06-1.48)	**0.009**	1.28 (1.08-1.51)	**0.004**
Corrected CT/PFA-100 (1), per 10 s	1.11 (1.07-1.15)	**<0.001**	1.04 (1.00-1.09)	**0.038**	1.03 (0.99-1.07)	0.200

P-values in bold denote p<0.05.

Table S9. Uni- and multivariable competing risk regression analyses of factors associated with hepatic decompensation/liver-related death including – among other parameters – CTP score, sodium, and creatinine **(model 1)** or UNOS MELD (2016) score, CS, and albumin, and **(model 2)** with removal of the primary aetiological factor/requirement of liver transplantation/non-liver-related death as competing risks.

Abbreviations: (a)SHR (adjusted) subdistribution hazard ratio; CRP C-reactive protein; CS clinical stage; CT clotting time; CTP Child-Turcotte-Pugh score; HVPG hepatic venous pressure gradient; PLT platelets; UNOS MELD (2016) score United Network for Organ Sharing model for end-stage liver disease (2016) score

Table S10

Patient characteristics	**Univariable**		**Model 1 (incl. CTP score, sodium, and creatinine)**		**Model 2 (incl. MELD, CS, and albumin)**	
	**SHR (95%CI)**	**p-value**	**aSHR (95%CI)**	**p-value**	**aSHR (95%CI)**	**p-value**
Age, year	1.03 (1.02-1.05)	**<0.001**	1.03 (1.01-1.05)	**<0.001**	1.03 (1.01-1.04)	**<0.001**
HVPG, mmHg	1.10 (1.07-1.12)	**<0.001**	1.05 (1.02-1.09)	**0.002**	1.04 (1.00-1.08)	**0.036**
CTP score						
A	1		1		-	-
B	2.36 (1.71-3.25)	**<0.001**	1.34 (0.89-2.00)	0.160	-	-
C	3.80 (2.48-5.82)	**<0.001**	1.79 (1.00-3.19)	**0.049**	-	-
UNOS MELD (2016) score, point	1.07 (1.05-1.11)	**<0.001**	-	-	1.01 (0.97-1.04)	0.770
CS						
CS 0	1		-	-	1	
CS 1	8.40 (2.02-34.90)	**0.003**	-	-	4.33 (1.02-18.31)	**0.047**
CS 2	16.00 (3.69-69.30)	**<0.001**	-	-	6.49 (1.42-29.67)	**0.016**
CS 3	14.40 (3.48-59.90)	**<0.001**	-	-	4.92 (1.12-21.74)	**0.035**
CS 4	21.30 (5.16-87.90)	**<0.001**	-	-	6.50 (1.46-28.90)	**0.014**
Sodium, mmol x L^{-1}	0.93 (0.90-0.97)	**<0.001**	0.99 (0.95-1.03)	0.670	-	-
Creatinine, mg x dL^{-1}	2.07 (1.26-3.41)	**0.004**	1.15 (0.68-1.94)	0.600	-	-
Albumin, g x L^{-1}	0.93 (0.91-0.96)	**<0.001**	-	-	0.98 (0.95-1.01)	0.170
CRP, mg x L^{-1}	1.87 (1.54-2.28)	**<0.001**	1.33 (1.03-1.72)	**0.031**	1.37 (1.05-1.79)	**0.020**
Corrected CT/PFA-100 (1), per 10 s	1.15 (1.09-1.21)	**<0.001**	1.07 (1.00-1.14)	**0.045**	1.05 (0.98-1.13)	0.150

P-values in bold denote $p<0.05$.

Table S10. Uni- and multivariable competing risk regression analyses of factors associated with liver-related death including – among other parameters – CTP score, sodium and, creatinine **(model 1)** or UNOS MELD (2016) score, CS, and albumin **(model 2)** with removal of the primary aetiological factor/requirement of liver transplantation/non-liver-related death as competing risks.

Abbreviations: (a)SHR (adjusted) subdistritubion hazard ratio; CRP C-reactive protein; CS clinical stage; CT clotting time; CTP Child-Turcotte-Pugh score; HVPG hepatic venous pressure gradient; PFA-100 Platelet Function Analyzer 100; UNOS MELD (2016) score United Network for Organ Sharing model for end-stage liver disease (2016) score

Table S11

Patient characteristics	<u>**Univariable**</u>		<u>**Multivariable**</u>	
	**SHR (95%CI)**	**p-value**	**aSHR (95%CI)**	**p-value**
CLIF-C ACLF-D score	1.11 (1.06-1.17)	**<0.001**	1.11 (1.06-1.16)	**<0.001**
CT/PFA-100, per 10 s	1.02 (0.99-1.04)	0.076	1.02 (0.99-1.04)	0.110

P-values in bold denote p<0.05.

Table S11. Uni- and multivariable competing risk regression analyses of factors associated with the development of acute-on-chronic liver failure (ACLF)/liver-related death including the CLIF-C ACLF-D score and PFA-100 with requirement of liver transplantation/non-liver-related death/etiological cure as competing risks in decompensated patients.

Abbreviations: ACLF acute-on-chronic liver failure; aSHR adjusted subdistribution hazard ratio; CLIF-C ACLF-D score Chronic Liver Failure Consortium acute-on-chronic liver failure Development score; CT clotting time; PFA-100 Platelet Function Analyzer 100

Table S12

Patient characteristics, n (%)	**Study cohort, n=688**
Any severe bleedings	87 (13%)
Severe portal-hypertensive bleedings	76 (11%)
Variceal bleedings	56 (8%)
Other gastrointestinal-PH-bleedings	20 (3%)
Any non-malignant thromboses	72 (10%)
Non-malignant portal vein thromboses	58 (8%)
Other Venous thromboses (deep vein thromboses, pulmonary embolisms, etc.)	7 (1%)
Arterial thromboses	7 (1%)

Categorical variables were reported as absolute (n) and relative frequencies (%).

Table S12. Bleeding/thrombotic events during follow-up.

Table S13

Patient characteristics	**Univariable**		**Model 1 (incl. CTP score, sodium, creatinine, and VWF-Ag)**		**Model 2 (incl. MELD, CS, albumin, and VWF-Ag)**	
	**B**	**p-value**	**B**	**p-value**	**B**	**p-value**
Age, year	0.635	**0.016**	0.326	0.273	0.383	0.183
Male sex	-14.016	**0.027**	-5.978	0.371	-5.344	0.412
BMI, kg x m^{-2}	1.000	0.075	0.428	0.495	0.610	0.323
Overweight[1]	6.411	0.285	-	-	-	-
Obesity[2]	11.566	0.111	-	-	-	-
Prediabetes[3]	6.188	0.425	-	-	-	-
Diabetes[4]	16.297	**0.032**	-1.036	0.888	-0.493	0.946
Arterial hypertension[5]	11.876	0.059	16.101	**0.019**	14.563	**0.034**
Hypertriglyceridemia[6]	-33.893	**0.004**	-15.080	0.213	-18.486	0.125
Hypercholesterolemia[7]	-7.567	0.431	-	-	-	-
HDL below threshold[8]	2.902	0.657	-	-	-	-
Statin use	0.611	0.970	-	-	-	-
Hepatic steatosis[9]	5.866	0.383	-	-	-	-
CTP score, point	5.295	**<0.001**	0.832	0.691	-	-
UNOS MELD (2016), point	2.216	**<0.001**	-	-	1.280	0.104
HVPG, mmHg	2.411	**<0.001**	0.404	0.514	0.515	0.411
dACLD (CS 2-4)	11.322	0.061	-	-	-18.650	**0.013**
Platelet count, G x L^{-1}	-0.404	**<0.001**	-0.366	**<0.001**	-0.374	**<0.001**
Haematocrit, %	-4.523	**<0.001**	-4.873	**<0.001**	-5.173	**<0.001**
Sodium, mmol x L^{-1}	0.630	0.378	0.965	0.264	-	-
Creatinine, mg x dL^{-1}	5.651	0.625	1.148	0.925	-	-
Albumin, g x L^{-1}	-1.410	**0.006**	-	-	0.491	0.443
CRP, mg x dL^{-1}	-6.351	0.218	-	-	-	-
VWF-Ag, %	0.004	0.870	-0.041	0.160	-0.045	0.124

[1]BMI ≥25 kg x m^{-2}
[2]BMI ≥30 kg x m^{-2}
[3]Fasting blood glucose 100-125mg x dL^{-1}; HbA1c 5.7-6.4%
[4]Fasting blood glucose >125mg x dL^{-1}, HbA1c ≥6.5%, or antidiabetic medication
[5]Blood pressure >140/90mmHg, or antihypertensive medication
[6]Triglycerides >150 mg x dL^{-1}
[7]Total cholesterol >200 mg x dL^{-1}
[8]<35mg x dL^{-1} for males and <39mg x dL^{-1} for females
[9]Biopsy-proven, controlled attenuation parameter >248dB x m^{-1}, or diagnosed by ultrasound
P-values in bold denote p<0.05.

Table S13. Simple and multiple linear regression analysis of factors associated with CT/PFA-100 including – among other parameters – either CTP score, serum sodium, creatinine, and VWF-Ag **(model 1)**, or UNOS MELD (2016) score, CS, albumin, and VWF-Ag **(model 2)**.

Abbreviations: ACLF acute-on-chronic liver failure; BMI body mass index; CRP C-reactive protein; CS clinical stage; CT clotting time; CTP Child-Turcotte-Pugh score; HVPG hepatic venous pressure gradient; PFA-100 Platelet Function Analyzer 100; UNOS MELD (2016) score United Network for Organ Sharing model for end-stage liver disease (2016) score; VWF-Ag von Willebrand Factor antigen

Table S14

Patient characteristics available in n=543 (79%) of patients	**Univariable**		**Model 1 (incl. CTP score, sodium, creatinine, and VWF-RCo)**		**Model 2 (incl. MELD, CS, albumin, and VWF-RCo)**	
	**B**	**p-value**	**B**	**p-value**	**B**	**p-value**
Age, year	0.589	**0.047**	0.426	0.208	0.499	0.127
Male sex	-12.713	0.071	-2.900	0.693	-3.706	0.606
BMI, kg x m^{-2}	1.072	0.083	-0.184	0.790	0.048	0.943
Overweight[1]	6.448	0.337	-	-	-	-
Obesity[2]	14.502	0.078	-	-	-	-
Prediabetes[3]	-0.109	0.159	-	-	-	-
Diabetes[4]	17.529	**0.037**	0.623	0.937	1.313	0.868
Arterial hypertension[5]	13.720	0.050	16.001	**0.036**	15.516	**0.041**
Hypertriglyceridemia[6]	-32.499	**0.009**	-11.466	0.381	-13.400	0.302
Hypercholesterolemia[7]	-10.417	0.334	-	-	-	-
HDL below threshold[8]	0.590	0.935	-	-	-	-
Statin use	13.444	0.462	-	-	-	-
Hepatic steatosis[9]	8.898	0.250	-	-	-	-
CTP score, point	4.352	**0.009**	1.851	0.410	-	-
UNOS MELD (2016), point	2.001	**0.002**	-	-	1.762	**0.037**
HVPG, mmHg	2.546	**<0.001**	1.504	**0.030**	1.468	**0.037**
dACLD (CS 2-4)	15.584	**0.020**	-	-	-15.018	0.082
Platelet count, G x L^{-1}	-0.401	**<0.001**	-0.356	**<0.001**	-0.371	**<0.001**
Haematocrit, %	-4.695	**<0.001**	-4.823	**<0.001**	-4.881	**<0.001**
Sodium, mmol x L^{-1}	1.052	0.177	1.419	0.118	-	-
Creatinine, mg x dL^{-1}	5.283	0.681	3.108	0.812	-	-
Albumin, g x L^{-1}	-1.115	**0.006**	-	-	0.340	0.617
CRP, mg x dL^{-1}	-6.217	0.270	-	-	-	-
VWF-RCo, %	-0.071	**0.021**	-0.175	**<0.001**	-0.189	**<0.001**

[1]BMI ≥25 kg x m^{-2}
[2]BMI ≥30 kg x m^{-2}
[3]Fasting blood glucose 100-125mg x dL^{-1}; HbA1c 5.7-6.4%
[4]Fasting blood glucose >125mg x dL^{-1}, HbA1c ≥6.5%, or antidiabetic medication
[5]Blood pressure >140/90mmHg, or antihypertensive medication
[6]Triglycerides >150 mg x dL^{-1}
[7]Total cholesterol >200 mg x dL^{-1}
[8]<35mg x dL^{-1} for males and <39mg x dL^{-1} for females
[9]Biopsy-proven, controlled attenuation parameter >248dB x m^{-1}, or diagnosed by ultrasound
P-values in bold denote p<0.05.

Table S14. Simple and multiple linear regression analysis of factors associated with CT/PFA-100 including – among other parameters – either CTP score, sodium, creatinine, and VWF-RCo **(model 1)**, or UNOS MELD (2016) score, CS, albumin, and VWF-RCo **(model 2)**.

Abbreviations: ACLF acute-on-chronic liver failure; BMI body mass index; CRP C-reactive protein; CS clinical stage; CT clotting time; CTP Child-Turcotte-Pugh score; HVPG hepatic venous pressure gradient; PFA-100 Platelet Function Analyzer 100; UNOS MELD (2016) score United Network for Organ Sharing model for end-stage liver disease (2016) score; VWF-RCo von Willebrand Factor ristocetin co-factor

Supplementary reference

1. Kuiper G, Houben R, Wetzels RJH, Verhezen PWM, Oerle RV, Ten Cate H, et al. The use of regression analysis in determining reference intervals for low hematocrit and thrombocyte count in multiple electrode aggregometry and platelet function analyzer 100 testing of platelet function. Platelets. 2017;28(7):668-75.
